# Tumor volume is a better predictor of post-operative wound complications compared to tumor size in soft tissue sarcomas of the proximal lower extremity

**DOI:** 10.1186/s13569-016-0041-7

**Published:** 2016-02-22

**Authors:** Michael Ziegele, David M. King, Manpreet Bedi

**Affiliations:** Department of Orthopaedic Surgery, Medical College of Wisconsin, 9200 West Wisconsin Ave, Milwaukee, WI 53226 USA; Department of Radiation Oncology, Medical College of Wisconsin, 9200 West Wisconsin Ave, Milwaukee, WI 53226 USA

## Abstract

**Background:**

Wide local excision with or without radiation therapy (RT) and chemotherapy is widely accepted as appropriate management for soft tissue sarcomas (STS) of the extremity. Although survival and local control rates are comparable to amputation, post-operative wound complications (WC) following limb salvage can result in significant morbidity for the patient. Certain risk factors such as location, pre-operative RT, and age have been shown to increase the risk of WCs. Somewhat surprisingly, size has not consistently been shown to impact WC rates. The goal of this study is to assess whether tumor volume, as opposed to the traditional measurement of the largest dimension in one plane, correlates with the development of post-operative WCs.

**Methods:**

Between 2000 and 2013, 81 patients with STS of the proximal lower extremity, buttock and pelvis were retrospectively identified from our prospective database. We reviewed the impact of patient, tumor, and treatment variables on postoperative WC. Predictors for WC were evaluated using the Fisher exact test for univariate analysis and logistic regression for multivariate analysis. Tumor volume was determined using the medical image merge (MIM) ^®^ software program (version 6.5.4, MIM Software, Cleveland, OH). Tumor size (diameter) was determined the historical way of measuring the widest dimension on the sagittal, coronal, and axial planes from the MRI scan at midplane.

**Results:**

The overall WC rate within 6 months of tumor resection was 32 %. WC were more likely to occur with larger tumor volumes (p = 0.015), although not with tumor diameters ≥10 cm (p = 0.55). Neither volume of subcutaneous fat (p = 0.34) or depth of the subcutaneous fat layer (p = 0.82) significantly impacted WC rates. Tumor proximity to skin surface also did not significantly impact WC risk (p = 0.73).

**Conclusions:**

Increase in tumor volume led to a higher risk of post-operative WCs. Assessing tumor volume may allow clinicians to better counsel patients on their risk of post-operative WCs. Tumor volume, as opposed to size alone, should be considered in future sarcoma outcome studies.

## Background

Approximately 12,000 patients in the United States each year are diagnosed with soft tissue sarcomas (STS), representing roughly 1 % of all adult malignancies [1]. In the past, due to concern for high local recurrence rates with local excision, amputation was routinely performed. However, there has been a transition in the management of STS of the extremity towards limb salvage resection in combination with radiation therapy (RT) with or without chemotherapy. This treatment approach has resulted in excellent local control rates [2, 3].

Despite advances in the treatment of STS, post-operative wound complications (WC) following surgical resection of the tumor remain an important source of morbidity for patients. WC have been reported in 16–56 % of surgical cases [4, 5], and can include complications such as seromas, hematomas, wound necrosis, wound dehiscence, cellulitis, and abscess formation.

Considerable research has been dedicated towards investigating risk factors for WC follow surgical resection of STS. Risk factors include, but are not limited to, location of disease, tumor size and proximity to skin, and timing of radiation. Tumor location is a strong predictive factor, with tumors located in the lower extremity (LE) experiencing higher rates of WC following surgery [6–10]. Korah et al. demonstrated that tumor location was the single most important risk factor for WC, and also reports a wound reoperation rate of 29 % for LE tumors vs. 4 % in upper extremity (UE) tumors [7].

Although location of the primary tumor has been consistently shown to impact the development of post-operative WCs, this has not been the case for tumor size. Moreover, most studies that evaluate size as a risk factor take into consideration the maximal dimension of the primary tumor. While the maximal measurement in one dimension gives an idea of tumor size, the overall size, or volume, difference between a 3 × 10 cm tumor and a 9 × 10 cm tumor is notable. There has been little information on the volume of the tumor and correlation to post-operative WCs.

Similar to tumor size, body mass index (BMI) and the amount of subcutaneous fat has not repeatedly been shown to influence WCs. This lack of consistency perhaps demonstrates that more than one measurement variable effects this outcome.

The objective of this study was to investigate additional variables that might impact post-operative WCs following surgical resection of STS. Specifically, utilizing a combination of volumetric and linear data gathered from preoperative MRIs, we determined if tumor volume and the ratio of subcutaneous (SC) fat, muscle and tumor impacted WC risk. To our knowledge the impact of SC fat on WC rates has never been investigated in STS, although high levels of SC fat have been linked with increased WC rates in other surgical procedures [11–13]. Additionally, the ability of tumor diameter to predict WC risk in comparison to tumor volume has never been investigated.

## Methods

This research was reviewed and approved by the Institutional Review Board (IRB) and all investigators completed training in both human research and patient privacy.

### Patient population

All patients with localized primary STS of the proximal LE, buttock, and pelvis who underwent wide surgical resection, with or without radiation, and/or chemotherapy between 2000 and 2013 were reviewed. Pelvic tumors in this study included sarcomas in the pelvis and buttock that were extra-peritoneal. No retroperitoneal or peritoneal tumors were included in this study. Additional exclusion criteria included metastatic disease on initial presentation, age <18 years old, STS of locations other than the proximal LE, buttock, or pelvis, recurrent or non-oncologic resection of sarcomas at first presentation to our sarcoma center, and small subcutaneous tumors. All patients underwent resection. Patients who had radiation (preoperative or post-operative) or no radiation were included in the study. Patients who did not have complete medical records including treatment information and a pathology report, and follow-up of less than 6 months were also excluded. Histopathologic types demonstrating rhabdomyosarcoma, extraosseous primitive neuroectodermal tumor, Kaposi’s sarcoma, angiosarcoma, and desmoid fibromatosis were also excluded.

Eighty-one patients were identified in our database who met the inclusion criteria. Patients were staged according to the 2009 American Joint Committee on Cancer (AJCC) system seventh edition.

### Treatment

All patients were discussed at a multidisciplinary tumor board consisting of surgical and musculoskeletal oncologists, medical and radiation oncologists, radiologists and pathologists. Treatment recommendations from this tumor board were presented to the patient.

### Radiotherapy and chemotherapy

Patients included in this study had either preoperative, post-operative or no RT. Those that had preoperative RT received a median dose of 50 Gy using 3D-conformal radiation or intensity-modulated radiotherapy (IMRT). Patients who received post-operative RT received a median dose of 60 Gy.

Chemotherapy was recommended and administered in patients who were typically <70 years of age, with large (>5 cm), deep, and high-grade lesions. Chemotherapy was a doxorubicin-ifosfamide based regimen given for 1–3 cycles based on clinical response and tolerance.

### Surgery

Limb-sparing resection was performed in all patients. Wide surgical resection was performed by fellowship trained musculoskeletal oncologists grossly through normal tissue planes with sacrifice of arteries or veins that were involved by tumor. Preservation of neurovascular structures was performed when possible. The goal of surgery was to achieve negative margins (R0). Vascular or reconstructive plastic surgeons were involved in cases that required vascular reconstruction, difficult wound closures and free flap reconstructions.

### Data collection

**Fig. 1:**
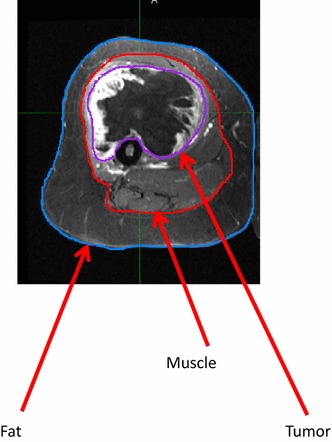
Forty-eight year old with a high grade myxofibrosarcoma of the proximal thigh. Axial (*A*), coronal (*B*) and sagittal (*C*) views with contours of fat (*blue*), muscle (*red*) and tumor (*purple*)

Pre-operative MRIs were downloaded into the medical image merge (MIM) ^®^ software program (version 6.5.4, MIM Software, Cleveland, OH). MRI’s were acquired within 1 month prior to limb-sparing resection. Post-operative MRI’s were also identified for linear measurements from the incision to the deep fascia. Using axial views, tumors were identified and contoured from proximal to distal tip, generating data on maximal tumor diameter and tumor volume. Volumes of subcutaneous fat and lean mass (including muscle, bone, and blood vessels) were gathered in a similar manner by contouring the structures from the proximal to distal boundaries of the tumor (Fig. 1). Tumor proximity to skin surface was measured from the point of maximal tumor diameter on the axial view to the future incision site determined from the patient’s post-operative MRI. Maximal subcutaneous fat layer depth was also measured in this manner, along the length of the tumor and directly adjacent to the future incision site.

Patient data, including age, sex, BMI, diabetic and smoking status, presence of cardiovascular disease, KPS score, tumor histology and stage/grade, use of flap reconstruction, and treatment with chemotherapy were gathered from the existing orthopedic oncology database of STS patients.

### Outcomes

Post-operative WCs were defined as by the Canadian Multicenter Trial and were recorded if they occurred within 6 months following limb-salvage surgery (Table 1). In general, patients who required re-operation, prolonged wound care or antibiotics after resection were considered WC [10].

**Table 1:** Canadian multicenter wound complication definition

Wound complication category	Definition
Surgical complications	Secondary operations required for wound treatment (debridement, secondary closure procedures such as rotationplasty, free flaps, or skin grafts) Invasive procedure required for wound care (drainage of hematoma, seroma, or infected wound collection) Deep wound packing required at any time (deep packing defined as packing deep to dermis in an area of dehisced wound) to an area of the wound measuring ≥2 cm in length
Non-surgical complications	Readmission to hospital for wound care Prolonged dressing changes, including packing of the wound for >6 weeks from wound break down Failure of epithelialization of skin graft by 4 weeks after surgery

### Statistical analysis

Statistical software MedCalc (Version 15.6; MedCalc Software bvba, Ostend, Belgium) was used for all data analysis. Clinical, pathologic and treatment characteristics for WC were assessed and summarized in Table 2. Wound outcome was a dichotomous variable. The fisher exact test was used for univariate analysis (UVA). If a variable had a *p* value of less than 0.25, then it was used in the multivariate model. A logistic regression analysis was used for multivariate analysis (MVA). For all analysis, type I error was maintained at 0.05 and all tests were two-sided.

**Table 2:** Clinical, pathologic, and treatment characteristics

Age (years)	
Mean	58.2
Median	57
Range	26–84
Gender	
F	30 (37 %)
M	51 (63 %)
Performance status (KPS)	
81–100	71(88 %)
≤80	10 (12 %)
Cardiovascular disease	
No	74 (91 %)
Yes	7 (9 %)
Diabetes	
No	72 (89 %)
Yes	9 (11 %)
Smoking history	
No	57 (70 %)
Yes	24 (30 %)
Stage	
I	20 (25 %)
II	10 (12 %)
III	51 (63 %)
Size	
<10 cm	38 (47 %)
≥10 cm	43 (53 %)
Location	
Proximal lower extremity	69 (85 %)
Buttock	8 (10 %)
Hip/pelvis	4 (5 %)
Grade	
Low	23 (28 %)
Intermediate	4 (5 %)
High	54 (67 %)
Histology	
Undifferentiated	10 (12 %)
Liposarcoma/leiomyosarcoma	31 (38 %)
Myxofibrosarcoma	11 (14 %)
Synovial	4 (5 %)
Spindle cell	13 (16 %)
Other	12(15 %)
Neoadjuvant chemotherapy	
No	25 (31 %)
Yes	56 (69 %)
Timing of RT	
No RT	8 (10 %)
Preoperative RT	70 (86 %)
Post-operative RT	3 (4 %)
Flap reconstruction	
No	50 (62 %)
Yes	31(38 %)

A receiver operative characteristic (ROC) curve was constructed and used to examine whether the volumetric data could differentiate between tumors that were predisposed to post-operative WCs versus those that were not predisposed to post-operative WCs.

## Results

Eighty-one patients with stage I–III STS of the LE and buttock underwent treatment at this institution and were eligible for the study. Sixty-nine (85 %) patients had tumors located in the proximal LE, eight (10 %) had tumors in the buttock, and four (5 %) had tumors located in the pelvic region. An overview of patient, tumor, and treatment characteristics can be found in Table 2. Median age at diagnosis was 57 (range: 26–84), and median follow-up was 1.7 years. All 81 patients underwent limb-salvage resection; no patient underwent an amputation at the time of definitive resection.

The overall WC rate within 6 months of tumor resection and as defined by the Canadian Multicenter Trial [10], was 32 % (26 of 81 patients). Of these, ten (38 %) patients developed wound dehiscence and necrotic wounds, ten (38 %) patients developed infection, five (20 %) patients had delayed wound healing, and one (4 %) patient developed a post-operative hematoma. Fifty-four (67 %) patients had high grade tumors, four (5 %) intermediate grade, and 23 (28 %) low grade. There was no significant difference in WC rates in patients with high grade (29.7 %) vs. low/intermediate grade (37.0 %) tumors (p = 0.61).

Median BMI of patients in our study was 28.8. WC occurred in 15 patients (37.5 %) with a BMI >28.8, and in eleven patients (28.2 %) with a BMI <28.8; this difference was not significant on UVA (p = 0.47) or MVA (p = 0.18). Similarly, when BMI was analyzed as a continuous variable, it did not significantly impact WC rates (OR 1.0448, 95 % CI 0.9746–1.1201, p = 0.22).

Forty-three patients had a tumor diameter of ≥10 cm. The WC rate was 39.5 % with tumors that were >10 cm compared to 23.6 % when the primary tumor measured <10 cm. This failed to reach statistical significance (p = 0.15). Increasing tumor volume, however, was associated with higher WC rates. The median tumor volume of patients in this study was 228.1 mL. WC occurred in 17/41 (41.5 %) patients with a tumor volume ≥228.1 mL, and in 9/40 (22.5 %) patients with a tumor volume <228.13 mL; p = 0.015). Increasing tumor volume was also significant when assessing this measure as a continuous variable (p = 0.015, OR 1.0010, 95 % CI 1.0001–1.0018). This finding held on MVA (p = 0.03, OR 1.0010, 95 % CI 1.0001–1.0018). A scatter plot depicting tumor size versus volume is located in Fig. 1.

The volume of subcutaneous fat did not significantly impact WC rates in this study (p = 0.34, OR 1.0002, CI 0.9998–1.0005). In addition, ratios comparing volumes of lean mass to subcutaneous fat (p = 0.69, OR 0.8683, 95 % CI 0.4306–1.7507) and volumes of subcutaneous fat to tumor (p = 0.55, OR 0.9822, 95 % CI 0.9257–1.0421) failed to reveal a significant relationship to WC on logistic regression. Depth of the subcutaneous fat layer at the incision site likewise did not impact WC rates.

Seventy (86 %) patients underwent preoperative RT, three (4 %) patients underwent postoperative RT, and eight (10 %) patients received both pre- and postoperative RT. There were no significant differences in WC rates between these groups. Fifty-six (69 %) patients received neoadjuvant chemotherapy, which had no significant impact on WC rates upon UVA (35.7 vs. 31.6 %, p = 0.44). No patient included in our study received adjuvant chemotherapy.

Primary closure was utilized for 50 (62 %) patients, while 31 (38 %) patients were selected for a vascularized flap closure with plastic surgery. Use of the flap closure was associated with higher WC rates (45 %) than primary closure (26 %), a difference which was significant on MVA (OR 3.6969, 95 % CI 1.2432–10.9938, p = 0.02). No other variables were significant on UVA or MVA (Table 3).

**Table 3:** UVA of WC variables

Variable	p value
BMI (>28/<28)	0.474
Neoadjuvant chemo (Y/N)	0.440
Cardiovascular disease (Y/N)	0.675
Diabetes (Y/N)	0.458
Smoking (Y/N)	0.798
Grade (high grade/non-high grade)	0.615
KPS score (>80/≤80)	0.278
Sex (M/F)	0.474
Tumor size (≥10 cm/<10 cm)	0.156
Flap closure (Y/N)	0.055

Local control in this cohort was 100 % and the distant metastasis rate was 30.8 %. Median survival was not met, but 2-year overall survival was 93.4 %. Median and 2-year progression-free survival was 66 months and 65 %, respectively. Similarly, median and 2-year distant metastasis-free survival was 66 months and 65 %, respectively.

## Discussion

While pre-operative RT combined with wide surgical resection of STS improves disease free survival and has become the standard in limb salvage care at many institutions, post-operative WCs remain a considerable source of morbidity for patients. Several studies have investigated variables that influence post-operative WCs, which include, but are not limited to tumor size and location, proximity to skin surface, radiation field size, and timing of radiation [4, 14–16].

Location of the primary tumor has consistently been shown to impact post-operative WCs. Moore et al. reports a 23 % difference in WC rate for proximal LE tumors and proximal UE tumors [6]. Tumors in the adductor compartment of the proximal thigh are particularly prone to WC, a trend likely explained by disruption of the lymphatic network of the LE which occurs during surgery [6]. For this study, we wanted to eliminate the confounding influence of tumor location on WC risk. Therefore, our cohort only included patients with tumors in the proximal LE, buttock, or pelvic area.

**Fig. 2:**
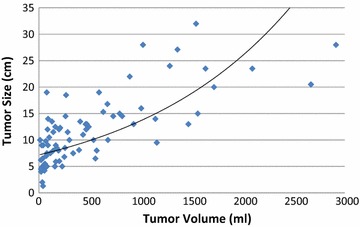
Tumor size vs. tumor volume scatter plot

**Fig. 3:**
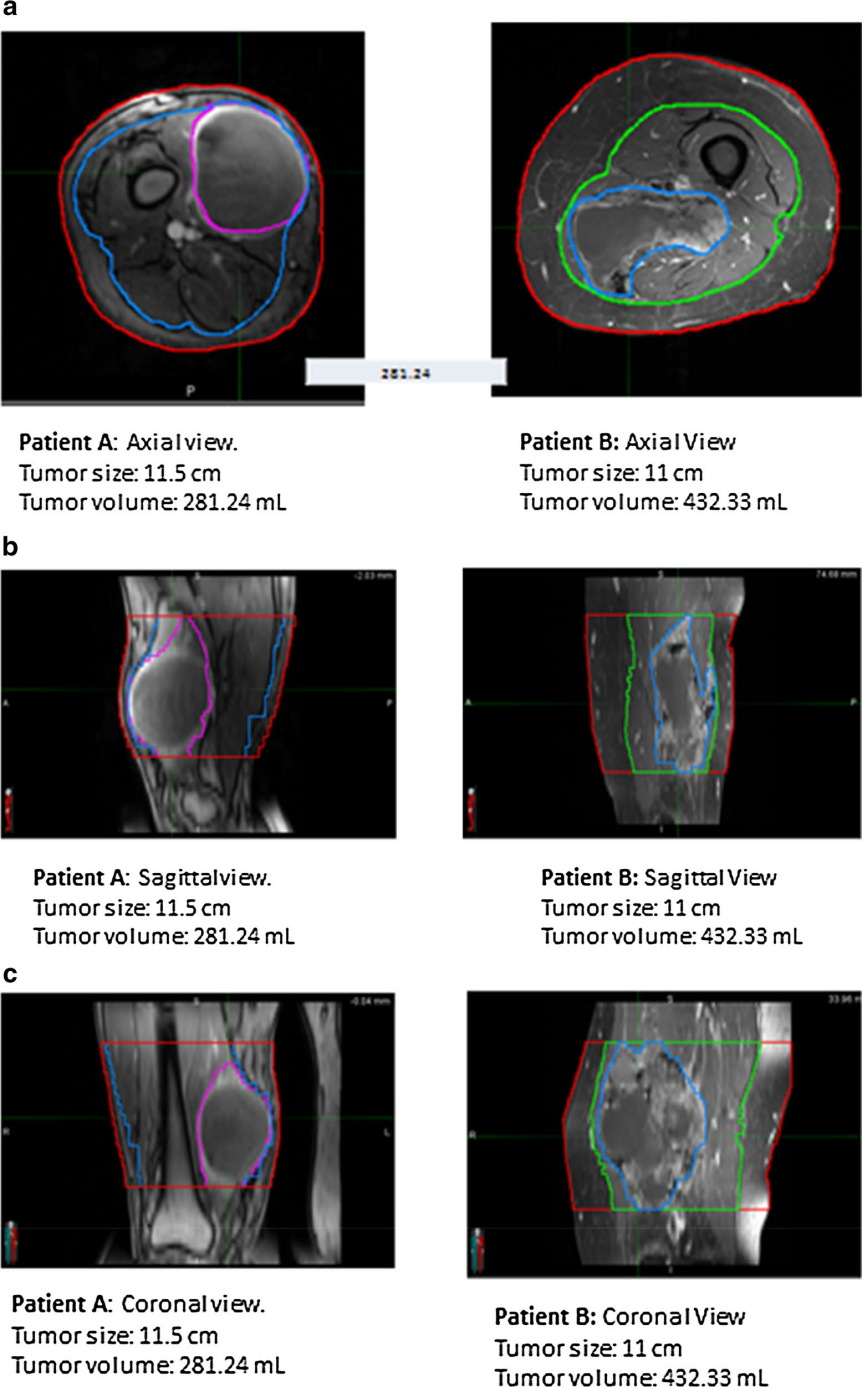
**a** Patient A and B’s Tumor size and corresponding volume on axial view. **b** Patient A and B’s tumor size and corresponding volume on sagittal view. **c** Patient A and B’s tumor size and corresponding volume on coronal view

Tumor size is a strong predictor of WC risk, a relationship which is demonstrated by multiple studies [4, 6, 7, 10, 17]. Tumors greater than 10 cm [4, 6, 10], 8 cm [7], and even 5 cm in diameter [18] have all been shown to be associated with higher WC risk. A probable explanation for the impact of tumor size on WC rates is that larger tumors generate a greater dead-space in the soft tissue following resection, predisposing the patient to seromas, hematomas, and infection. Our study did not demonstrate that tumors greater than 10 cm in diameter were associated with a statistically significant higher risk of WCs. We did however find a statistically significant relationship between tumor volume and WC rate, establishing that increasing tumor volume predicts a greater risk of postoperative WC. This finding first corroborates prior research on the importance of tumor size as a risk factor for postoperative WC. More notably however, it also suggests that tumor volume, rather than tumor diameter or cross-sectional area, is a more powerful predictor of postoperative WC risk. Moreover, as conveyed in Fig. 2, there seems to be little relationship between size and volume, most likely because STS are often not spherical, but have an irregular shape. Thus, as mentioned above, maximal dimension may only provide one component influencing WC, and it is truly the volume that impacts this outcome. By measuring a 3-dimensional tumor in only one plane it is easy to over or under estimate true tumor burden, thereby misjudging the soft-tissue defect which will be left post-resection along with the associated WC risk (Fig. 3a–c). Work by Geller et al. has previously demonstrated that tumor volume is associated with WC risk [5]. In this study however, tumor volume was measured from final gross pathology specimens and was also not compared to tumor diameter or cross sectional area. To our knowledge, our study is the first to assess if tumor volumes contoured from preoperative MRIs predict postoperative WC risk. It also for the first time demonstrates that volumetric measurements may be more representative of tumor size than measuring tumor diameter, a finding which has implications for predicting WC risk, as well as potentially other treatment outcomes, preoperatively.

With the expanding obesity epidemic in the United States, the impact of subcutaneous fat on WC risk is a crucial area of investigation in STS. BMI has failed to demonstrate a significant association with WCs in prior studies [6], as well as our own. However, BMI fails to take into account factors such as gender differences in adipose tissue distribution and also levels of lean muscle mass which may weaken any association with WC risk. Levels of subcutaneous fat tissue surrounding the incision site has yet to be investigated in STS, although it has been demonstrated to impact WC rates in other arenas of surgery. In one study of cervical spine fusions, patients with subcutaneous fat layer depths of >40 mm were at significantly higher risk of surgical site infection (18.2 %) than patients with thinner layers of <20 mm (5.2 %) and <10 mm (2.3 %) [17]. Similar results have been published in gynecologic and general surgery, indicating that the thickness of SC fat surrounding the incision site impacts WC risk [11–13, 19, 20]. Increased SC fat thickness necessitates a longer surgical incision, as well as increased retraction which may lead to tissue necrosis at the operative site. A thicker subcutaneous layer also increases wound tension at closure, placing the patient at increased risk for wound dehiscence and also reducing tissue microperfusion and oxygen availability to the wound [21]. In contrast to the aforementioned research, our study did not demonstrate a significant relationship between WC risk and either subcutaneous fat volume or depth of the adipose layer adjacent to the incision. One limitation to our study which may have impacted results is that a nine of the preoperative MRIs had small sections of peripheral tissue cut off, not allowing us to fully contour fat and muscle layers. Additionally, our measurement of subcutaneous fat volume included adipose tissue from the entire circumference of the leg, not just surrounding the incision site. An avenue for future research may be measurement of the SC fat volume surrounding only the leg compartment specific to the tumors location.

Reconstruction of surgical wounds with vascularized flaps is utilized as an alternative to primary closure following resection of STS. It is intended to lower WC risk through minimizing residual dead space and also substituting previously irradiated soft tissues with healthy and well-vascularized tissue from a donor site. The literature returns mixed results on the impact of plastic reconstruction on WC risk; some studies conclude that it lowers risk [22], others find that it increases it [4], while yet others demonstrate no impact [6]. Analysis of our patient cohort indicates that reconstruction with vascularized flaps was a statistically significant risk factor for WC. It should be noted however that there is likely a strong element of selection bias in this finding. Patients selected for immediate flap reconstruction at our institution were often those felt to be at higher risk for postoperative WC. These included patients with greater tumor volumes, vascular involvement of their tumor, and host comorbidities which may have increased their risk for WCs.

The association between preoperative RT and increased WC risk has been well established. O’Sullivan et al. reported an 18 % increase in WC rate with preoperative RT in comparison to postoperative RT, a finding which has been replicated by additional studies [6, 23]. Timing of RT was not a significant risk factor for WC in our study; however this is not surprising as the vast majority of our patients underwent preoperative RT. Similarly, the administration of neoadjuvant chemotherapy did not predispose patients to WC in our study. This is a finding which is consistent with other published work.

Baldini et al. describes that tumor proximity to skin surface impacts WC risk, reporting that tumors located <3 mm from the skin surface increase risk for WC (OR 3.9) [4]. This finding however has not been replicated in additional research [6], and is also not shown in our findings. Timing of radiotherapy in our study may be a factor in this negative result. Baldini et al. suggests that preoperative RT of superficial tumors delivers an unavoidably high radiation dose to the surgical flaps used in wound closure, increasing the risk for WC. While the majority of patients in our study received preoperative RT (86 %), some did undergo postoperative RT or no RT at all, which may have impacted results. Another possibility is that our small sample size limited our ability to find a significant relationship.

One weakness of our study was our small sample size. STS are exceedingly rare cancers, and by restricting our study population to only patients with tumors in the proximal LE, buttock, or pelvis, we limited our sample size. This could potentially explain why some of our results did not reach the level of statistical significance. For instance, prior research has demonstrated diabetes to be powerful risk factor for WC following resection of STS [4, 6]. In our study there were large differences in WC risk for patients with diabetes (44.4 vs. 30.6 %). This difference was not significant however, which may be due to our analysis being underpowered. Additional limitations to our study include the retrospective nature of the study and the inherent biases which come with this design, as well as the limited field of view on nine of the preoperative MRIs which limited our ability to accurately contour structures. Another limitation of the study is the potential error in the volume measurements. Contouring structures on MRI scans is a very operator dependent process, and results may vary from person to person based on contouring technique. We attempted to limit the potential impact of this by having all measurements acquired by a single researcher. Although all data was generated by a single researcher in our study, the intra and inter-observer variability associated with the volume measurement technique is unknown and attempts to replicate this research in the future may be prone to this bias.

## Conclusions

In this study, it was tumor volume, more than size that impacted the development of post-operative WCs. Our study demonstrates that gathering data on tumor volume, rather than diameter, may be a more accurate means of predicting WC risk. Tumor volume measurements could allow clinicians to more accurately counsel patients regarding their risk of WCs and also arrange for more aggressive follow-up and prophylactic regimens to combat the risk of WC. Further investigations on the relationship between volumetric parameters and treatment outcomes are warranted.

## Abbreviations

BMI: body mass index
CI: confidence interval
Gy: gray
MIM: medical image merge
MVA: multivariate analysis
STS: soft tissue sarcomas
UVA: univariate analysis
WC: wound complication
